# Supplementary material for: Prevalence and regional disparities of undiagnosed diabetes mellitus in Bangladesh: Results from the Bangladesh Demographic and Health Survey data
Source: PLoS One. 2025 Apr 2;20(4):e0321069. doi: 10.1371/journal.pone.0321069 (PMC11964274; doi:10.1371/journal.pone.0321069)
Supplement: S1 File — S1 Table. Results from a multivariable multilevel logistic regresson model. S2 Table. AIC value of Multinomial logistic regression model. S1 Fig. Random forest model. S2 Fig. Mosaic plot with residenital regions to investigate potential confounders. S3 Fig. Dagitty plot to investigate potential confounders. (PDF) [file pone.0321069.s001.pdf]

Supporting Information

Table of Contents

S1 TABLE. 1

S1 FIG. 3

S2 TABLE. 4

S2 FIG. 4

S3 FIG. 7

S1 Table.

We performed a sensitivity analysis using a multilevel multinomial regression model implemented with the brms package in R. The model includes geographic residence as a random effect, and the results are provided in the Appendix. This multilevel multinomial logistic regression approach was used to investigate regional disparities in the prevalence of diagnosed and undiagnosed diabetes compared to no diabetes. Each parameter in the results is summarized by the mean (Estimate) and standard deviation (Est.Error) of the posterior distribution, along with two-sided 95% Credible Intervals (l-95% CI and u-95% CI) derived from quantiles.

```
>  library(brms)
>
>  model3 <- brm(diabetes_status ~ Gender+Agegroup+Hypertension+BMIgroup+Residence + (1 | Residence_region),
+                family = categorical(), cores = 4, data = df2)
Compiling Stan program...
Trying to compile a simple C file
Start sampling
Warning messages:
1: There were 16 divergent transitions after warmup. See
https://mc-stan.org/misc/warnings.html#divergent-transitions-after-warmup
to find out why this is a problem and how to eliminate them.
2: Examine the pairs() plot to diagnose sampling problems

tart sampling
>  summary(model3)
 Family: categorical
  Links: muDiagnosed = logit; muUndiagnosed = logit
Formula: diabetes_status ~ Gender + Agegroup + Hypertension + BMIgroup + Residence + (1 | Residence_region)
   Data: df2 (Number of observations: 11911)
  Draws: 4 chains, each with iter = 2000; warmup = 1000; thin = 1;
         total post-warmup draws = 4000

Multilevel Hyperparameters:
~Residence_region (Number of levels: 4)
                            Estimate Est.Error l-95% CI u-95% CI Rhat Bulk_ESS Tail_ESS
sd(muDiagnosed_Intercept)       0.14      0.23     0.00     0.65 1.00     1217      923
sd(muUndiagnosed_Intercept)     0.57      0.36     0.19     1.58 1.00     1075     1564

[illegible]

Regression Coefficients:
                              Estimate Est.Error l-95% CI u-95% CI Rhat Bulk_ESS Tail_ESS
muDiagnosed_Intercept            -4.34      0.20    -4.73    -3.97 1.00     2209     1865
muUndiagnosed_Intercept          -3.08      0.33    -3.71    -2.28 1.00     1043     1019
muDiagnosed_GenderMale           -0.12      0.12    -0.35     0.11 1.00     4482     2904
muDiagnosed_Agegroup18M29        -2.02      0.37    -2.76    -1.34 1.00     2985     2451
muDiagnosed_Agegroup40M49         0.65      0.18     0.30     1.01 1.00     2497     2519
muDiagnosed_Agegroup50M59         1.24      0.18     0.90     1.60 1.00     2740     2901
muDiagnosed_Agegroup60M69         1.31      0.19     0.94     1.69 1.00     2685     2551
muDiagnosed_Agegroup70P           0.77      0.25     0.29     1.24 1.00     2939     2891
muDiagnosed_HypertensionYes       0.63      0.12     0.40     0.87 1.00     4567     2670
muDiagnosed_BMIgroupObese         0.88      0.21     0.45     1.27 1.00     4250     3194
muDiagnosed_BMIgroupOverweight    0.67      0.13     0.42     0.92 1.00     4056     2790
muDiagnosed_BMIgroupUnderweight  -1.67      0.33    -2.36    -1.07 1.00     4682     2294
muDiagnosed_ResidenceCity         0.60      0.17     0.25     0.92 1.00     3937     2545
muDiagnosed_ResidenceSemiMUrban   0.38      0.13     0.11     0.62 1.00     4762     3065
muUndiagnosed_GenderMale          0.11      0.08    -0.05     0.26 1.00     5069     3105
muUndiagnosed_Agegroup18M29      -0.37      0.11    -0.60    -0.14 1.00     3100     2801
muUndiagnosed_Agegroup40M49       0.12      0.12    -0.12     0.36 1.00     3108     2611
muUndiagnosed_Agegroup50M59       0.30      0.13     0.04     0.57 1.00     3401     2974
muUndiagnosed_Agegroup60M69       0.03      0.16    -0.28     0.34 1.00     3523     2598
muUndiagnosed_Agegroup70P         0.25      0.17    -0.10     0.58 1.00     3913     3159
muUndiagnosed_HypertensionYes     0.34      0.09     0.15     0.52 1.00     3955     2360
muUndiagnosed_BMIgroupObese       0.63      0.17     0.30     0.97 1.00     4349     2857
muUndiagnosed_BMIgroupOverweight  0.48      0.10     0.29     0.68 1.00     4248     2523
muUndiagnosed_BMIgroupUnderweight -0.20     0.13    -0.45     0.05 1.00     4121     2789
muUndiagnosed_ResidenceCity       0.35      0.13     0.10     0.59 1.00     4124     2994
muUndiagnosed_ResidenceSemiMUrban 0.20      0.09     0.02     0.38 1.00     4049     2718
```

S1 Fig.

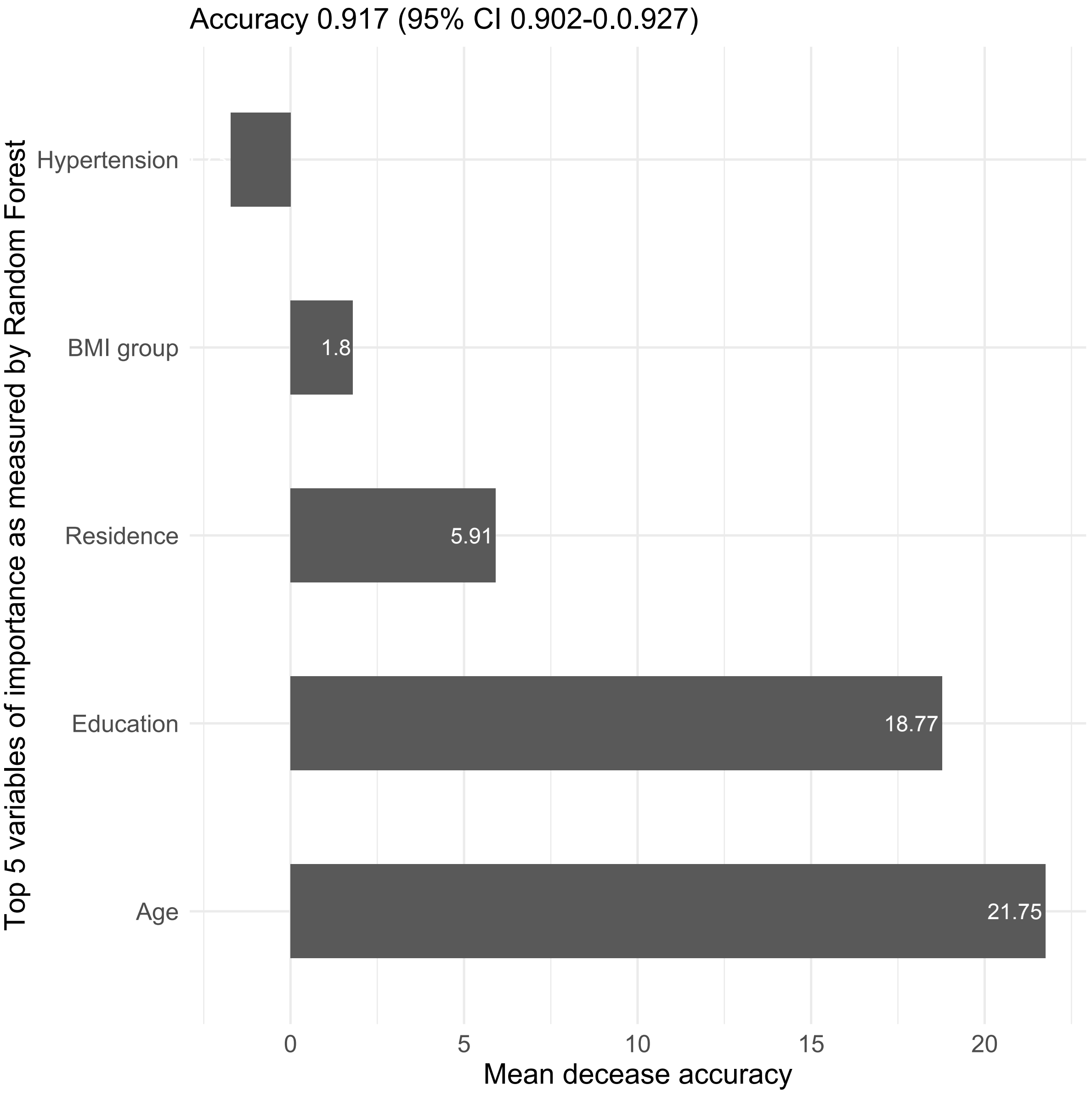

Accuracy 0.917 (95% CI 0.902-0.0.927)
Top 5 variables of importance as measured by Random Forest
Hypertension
BMI group
1.8
Residence
5.91
Education
18.77
Age
21.75
0
5
10
15
20
Mean decease accuracy

S2 Table.

```
> summary(multiresult)
Call:
multinom(formula = Diabetes1 ~ Gender + Agegroup + Hypertension +
    BMIgroup + Residence, data = df2)

Coefficients:
            (Intercept) GenderMale Agegroup30-39 Agegroup40-49 Agegroup50-59 Agegroup60-69 Agegroup70+ HypertensionYes
Diagnosed     -5.708011 -0.1231161      1.992447     2.6443505     3.2303444     3.3039814   2.7802780       0.6333381
Undiagnosed   -3.074847  0.1077012      0.359385     0.4796769     0.6621294     0.3968291   0.6434741       0.2921789
            BMIgroupObese BMIgroupOverweight BMIgroupUnderweight ResidenceRural ResidenceSemi-Urban
Diagnosed       0.8915909          0.6705442          -1.6371817     -0.6055044          -0.2303191
Undiagnosed     0.6340693          0.4869711          -0.1745151     -0.4182587          -0.1917122

Std. Errors:
            (Intercept) GenderMale Agegroup30-39 Agegroup40-49 Agegroup50-59 Agegroup60-69 Agegroup70+ HypertensionYes
Diagnosed     0.3708767 0.11842889     0.3601324     0.3553867     0.3565843     0.3618524   0.3972559      0.11896090
Undiagnosed   0.1468719 0.08279015     0.1166477     0.1251332     0.1381854     0.1617927   0.1758262      0.09245303
            BMIgroupObese BMIgroupOverweight BMIgroupUnderweight ResidenceRural ResidenceSemi-Urban
Diagnosed       0.2067841         0.12809542           0.3289946      0.1718229           0.1814698
Undiagnosed     0.1696648         0.09646042           0.1254520      0.1278813           0.1364852

Residual Deviance: 7577.43
AIC: 7629.43
```

Null model:

```
> multiresult1
Call:
multinom(formula = Diabetes1 ~ 1, data = df2)

Coefficients:
            (Intercept)
Diagnosed     -3.489385
Undiagnosed   -2.794737

Residual Deviance: 8141.081
AIC: 8145.081
```

The AIC value of 7629.43 suggests that the model achieves a reasonable balance between fit and complexity. Furthermore, when compared to the null model, the reduction in the AIC value indicates that this model provides a better fit to the data.

The residual deviance of 7577.43 also points to a good fit of the model to the data. Its significance can be further assessed by comparing it to the null deviance, which helps determine the extent to which the model improves over a baseline model with no predictors.

S2 Fig.

The plots depict the distribution of various demographic, health, and socioeconomic factors across residential regions (City, Rural, and Semi-Urban). Key observations include:

- Older populations (60+ years) are more concentrated in rural and semi-urban areas, likely due to aging in place and the migration of younger generations to urban centers.
- Urban areas exhibit a higher prevalence of hypertension, potentially driven by lifestyle factors such as consumption of processed foods, physical inactivity, and stress.
- The prevalence of obesity and overweight individuals is greater in urban regions, likely influenced by dietary habits, sedentary lifestyles, and socioeconomic status.
- In contrast, underweight individuals are more common in rural areas, possibly linked to lower income levels and nutritional deficiencies.

Regarding education:

- Higher education levels (college or above) are significantly associated with urban areas, likely due to better access to universities and educational institutions.
- Primary or no education is more prevalent in rural regions, highlighting disparities in access to educational resources.

- In terms of employment and income, urban areas have higher employment rates, reflecting greater job availability in industries, services, and corporate sectors.
- Rural areas show a higher proportion of individuals who are "not working," which may include agricultural laborers or unemployed individuals.
- Urban residents are more likely to fall into the "rich" category, reflecting better income opportunities and higher living costs.
- Rural areas have a higher percentage of "poor" individuals, consistent with limited job opportunities and lower wages.

These patterns underscore significant disparities in health, education, employment, and income across residential regions.

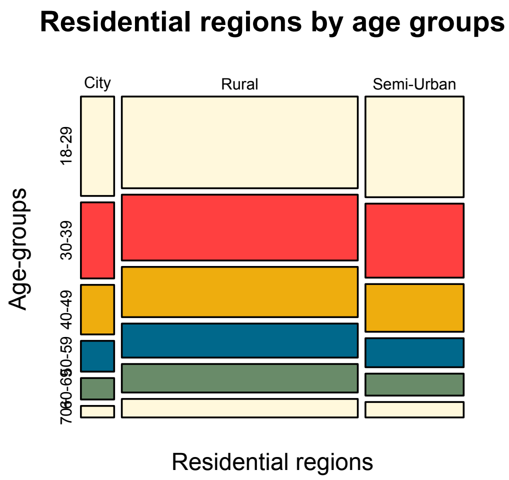
Residential regions by age groups
City
Rural
Semi-Urban
Age-groups
18-29
30-39
40-49
Residential regions

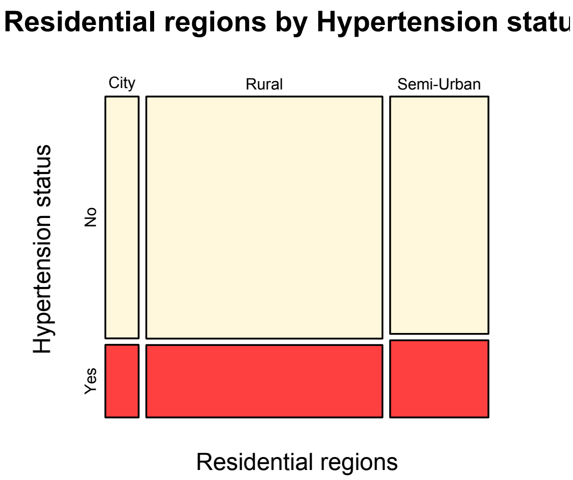
Residential regions by Hypertension statu
City
Rural
Semi-Urban
Hypertension status
No
Yes
Residential regions

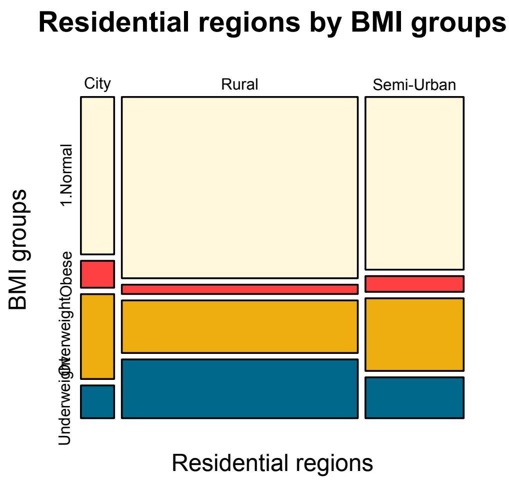
Residential regions by BMI groups
City
Rural
Semi-Urban
BMI groups
1.Normal
Obese
Underweight
Residential regions

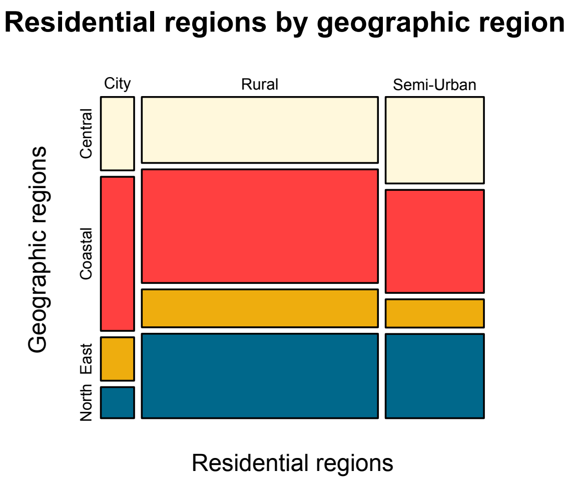
Residential regions by geographic region
City
Rural
Semi-Urban
Geographic regions
Central
Coastal
East
North
Residential regions

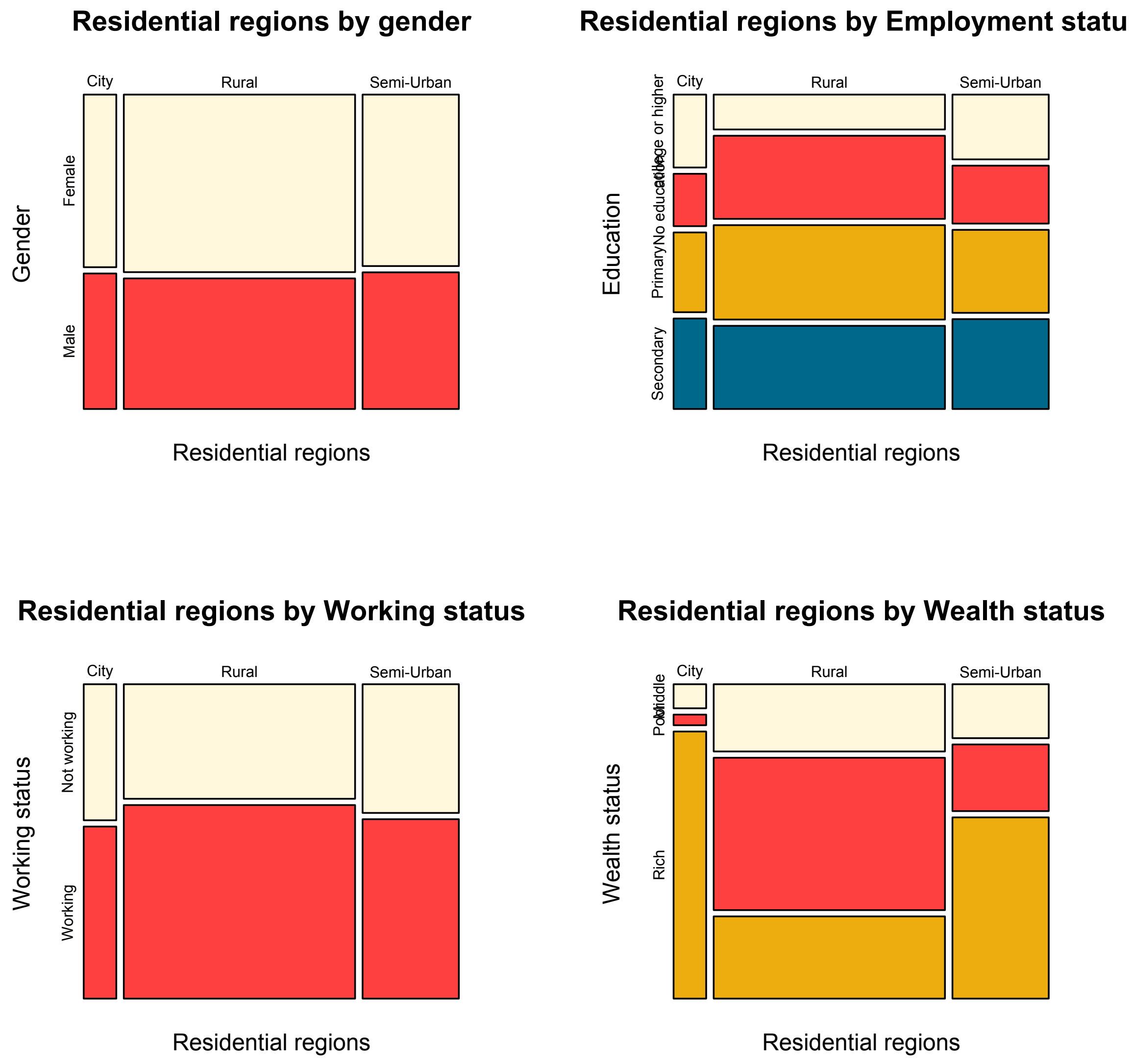

S3 Fig.

The Directed Acyclic Graph (DAG) (https://www.dagitty.net/dags.html#) illustrates potential causal relationships between various factors and diagnosed hypertension. The primary outcome is diagnosed hypertension, with coastal residency as the main exposure variable. Factors such as age, sex, education, marital status, smoking status, physical activity, BMI, occupation, and comorbid conditions with hypertension serve as potential confounders, mediators, or effect modifiers influencing the link between coastal residency and hypertension.

Arrows in the DAG indicate different types of relationships:

- **Solid black arrows** denote direct causal relationships, e.g., an arrow from "Age" to "Diagnosed Hypertension" indicates that age directly affects hypertension risk.
- **Dashed purple arrows** signify potential confounding or mediating influences. For instance, an arrow from "Smoking Status" to both "Diagnosed Hypertension" and "Coastal Residency" suggests that smoking may confound the relationship between exposure and outcome.
- **Dotted green arrows** represent possible effect modification. For example, an arrow from "Education" to the relationship between "Coastal Residency" and "Diagnosed Hypertension" suggests that education might alter the impact of coastal residency on hypertension risk.

  A minimum set of variables, including age, sex, BMI, and coastal residency, is sufficient to explore the relationship between coastal residency and diagnosed hypertension. Statistical analysis was then conducted to estimate the effect size and significance of coastal residency on hypertension, adjusting for confounders and mediators.

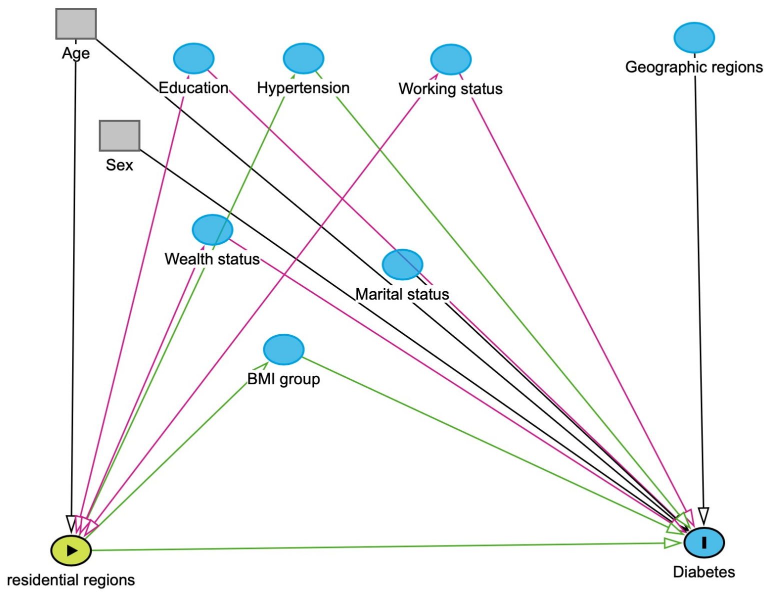

Figure. DAG demonstrating causal relationships and potential biasing pathways affecting the association between diabetes and residency (produced using DAGitty V.2.3 software).
