# Supplementary material for: Prevalence and regional disparities of undiagnosed diabetes mellitus in Bangladesh: Results from the Bangladesh Demographic and Health Survey data
Source: PLoS One. 2025 Apr 2;20(4):e0321069. doi: 10.1371/journal.pone.0321069 (PMC11964274; doi:10.1371/journal.pone.0321069)
Supplement: Attachment — Submitted filename: Reviewers report 2_PONE.docx [file pone.0321069.s004.docx]

Date: February 11, 2025

To

Editor

Plos One

RE: Prevalence and regional disparities of Undiagnosed Diabetes Mellitus in Bangladesh: Results from the Bangladesh Demographic and Health Survey data 
PONE-D-23-27830R1

Dear Editor:

Thank you very much for your editorial suggestions and the reviewers’ comments. They were accommodating. Please find enclosed an itemized list of responses along with the revised manuscript.

In our response to the reviewer, we used regular font for the comments/questions by the referees and regular, bold font for our responses, which are shown immediately following the questions/comments.

Thank you once again for the opportunity to submit a revised manuscript.

Ahmed Hossain, PhD

Professor, Health Care Management, University of Sharjah, Sharjah, UAE.

and

Director, Global Health Institute

North South University.

**E-mail:** [**ahossain@sharjah.ac.ae**](mailto:ahossain@sharjah.ac.ae)

Table of Contents

[Reviewer #3:	2](#_Toc190162966)

[Reviewer #4:	4](#_Toc190162967)

# Reviewer #3:

Unfortunately, I did not participate in the first round of evaluations, so my observations were not incorporated until this second round, which should contain specific aspects and not be as generic as the following:

Consideration 1:

The RR is usually defined as the incidence rate of the outcome in the treatment or exposed group divided by the incidence rate of the outcome in the control or non-exposed group. The RRR is defined as a risk reduction. These indicators or effect measures are used in longitudinal studies, not in cross-sectional studies.

**Authors: Multinomial logistic regression is a powerful statistical tool used in cross-sectional studies to model outcomes with more than two categories, allowing researchers to estimate relative risk ratios (RRRs) for each category relative to a baseline category (Camey et al.). The multinomial logit model is often preferred over traditional regression approaches in cross-sectional studies due to its theoretical and empirical advantages (Gensch et al., Dixit et al.). It is crucial to avoid using odds ratio (OR) as a proxy for RRR or PR in multinomial logistic regression, as this can lead to significant misinterpretations (Camey et al. and Blizzard et al.). For multinomial outcomes the OR must not be used as an approximation of the RR or PR, since this may lead to incorrect conclusions (Camey et al.).**

**Gensch, D., & Recker, W. (1979). The Multinomial, Multiattribute Logit Choice Model. Journal of Marketing Research, 16, 124 - 132. https://doi.org/10.1177/002224377901600117.**

**Dixit, S., Kumar, B., Singh, A., & Ashoka, R. (2015). An Application of Multinomial Logistic Regression to Assess the Factors Affecting the Women to Be Underweight and Overweight: A Practical Approach. -. International Journal of Health Sciences and Research, 5, 11-17.**

**Camey, S., Torman, V., Hirakata, V., Cortes, R., & Vigo, Á. (2014). Bias of using odds ratio estimates in multinomial logistic regressions to estimate relative risk or prevalence ratio and alternatives.. Cadernos de saude publica, 30 1, 21-9 . https://doi.org/10.1590/0102-311X00077313.**

**Blizzard, L., & Hosmer, D. (2006). The Log Multinomial Regression Model for Nominal Outcomes with More than Two Attributes. Biometrical Journal, 49. https://doi.org/10.1002/bimj.200610377.**

Consideration 2:

In logistic regression (binomial or multinomial), the coefficients of the logit (β), if they are exponents (expβ), are interpreted as ORs. These ORs are used to measure the association between risk factors and the prevalence of a condition. Although ORs are not exactly the same as prevalence ratios (PRs), they can be interpreted in a similar way to understand the relationship between variables.

For these reasons, it is preferable to use OR rather than RRR.

On other hand, ORs in a prevalence study do not allow the following to be said:

“Education played a significant role: individuals with primary and secondary education had 52% (RRR = 1.52, 95% CI = 1.07-2.15) and 63% (RRR = 1.63, 95% CI = 1.12-2.38) higher risk of undiagnosed diabetes, respectively, compared to those with no formal education”.

However, the following paragraphs are more appropriate:

• Education played a significant role: individuals with primary and secondary education had 52% (OR = 1.52, 95% CI = 1.07-2.15) and 63% (OR = 1.63, 95% CI = 1.12-2.38) increased odds of having undiagnosed diabetes, respectively, compared to those with no formal education.

• The odds of having undiagnosed diabetes were 1.5 times (OR = 1.52, 95% CI = 1.07-2.15) and 1.6 times (OR = 1.63, 95% CI = 1.12-2.38) higher for those with primary and secondary education, respectively, compared with those with no formal education.

• Education played a significant role: individuals with primary and secondary education had 52% (OR = 1.52, 95% CI = 1.07-2.15) and 63% (OR = 1.63, 95% CI = 1.12-2.38) higher prevalence of undiagnosed diabetes, respectively, compared to those with no formal education.

**Authors: We sincerely appreciate your valuable suggestion. For consistency and clarity, we have retained the presentation of results as Relative Risk Ratios (RRR) derived from the multinomial logistic regression model.**

Other paragraphs need to be improved:

“We found hypertension is strongly associated with an increased risk of both diagnosed and undiagnosed diabetes”

This is because the study did not have a follow-up period, and it is not possible to calculate incidence (risk) rates. The following paragraph is more appropriate:

“We found hypertension is strongly associated with both diagnosed and undiagnosed diabetes”

**Authors: Thank you for your wonderful suggestion. We corrected it accordingly.**

It is recommended that authors read the following document in order to use the terms correctly:

Hsueh, L., Wu, W., Hirsh, A. T., de Groot, M., Mather, K. J., & Stewart, J. C. Undiagnosed diabetes among immigrant and racial/ethnic minority adults in the United States: National Health and Nutrition Examination Survey 2011-2018. Annals of Epidemiology 2020, 51: 14–19. https://doi.org/10.1016/j.annepidem.2020.07.009

**Authors: Many thanks for your reference. The article that you recommended has outcome with two categories, but we have an outcome with three categories.**

Additionally, other limitations must be included. Firstly, glucose levels were measured using capillary blood, and although a conversion to plasma glucose was performed, this method may still introduce some inaccuracies. Second, a single blood glucose measurement is not sufficient to diagnose diabetes. It is possible that some people diagnosed with diabetes may not have the condition on a second measurement of fasting plasma glucose (false positive) and, conversely, some people who do have diabetes may be missed (false negative).

**Authors: Thanks. The article incorporates the limitations suggested in our discussion.**

**Glucose levels were measured using capillary blood, and while a conversion to plasma glucose was applied, this approach may still result in some inaccuracies. Moreover, a single blood glucose measurement to diagnose diabetes in the study can lead to diagnostic errors due to its potential for false positives and false negatives.**

# Reviewer #4:

While the manuscript addresses a critical and important topic, it requires substantial revisions before it can be considered for publication. My major comments are outlined below:

Methods:

• Compared to the standard multinomial logistic regression model, multilevel multinomial logistic regression analysis offers significant advantages. It reduces parameter overestimation and provides more accurate estimates, particularly in hierarchical data structures like the DHS survey. The authors are encouraged to consider this approach or discuss why they opted for the current method.

**Authors: Thank you very much for your wonderful suggestion. We applied the multilevel multinomial logistic regression model and the results are shown in sensitivity analysis.**

**We conducted a sensitivity analysis using a multilevel multinomial regression model implemented with the brms package in R. The model applies the geographic residence as the random effect and results are presented in the Appendix. The multilevel multinomial logistic regression model was employed to examine regional disparities in the prevalence of diagnosed and undiagnosed diabetes compared to no diabetes. In the results, each parameter is summarized using the mean (Estimate) and standard deviation (Est.Error) of the posterior distribution, along with two-sided 95% Credible Intervals (l-95% CI and u-95% CI) derived from quantiles.**

**The findings on regional disparities indicate that individuals in city areas are exp(0.60) = 1.822 times more likely to have diabetes compared to those in rural regions. The one-sided 95% credibility interval does not include zero, suggesting statistical significance. Additionally, city areas show that individuals are exp(0.35) = 1.42 times more likely to have undiagnosed diabetes compared to rural regions. Similar results were observed when using a multinomial regression model.**

• To assess the stability of a multinomial logistic regression model applied to cross-sectional data, various methods focusing on model assumptions, goodness-of-fit, and predictive performance across data subsets can be employed. This assessment is missing in the manuscript. Additionally, the results of multicollinearity checks were not reported. Addressing these aspects would strengthen the validity of the findings.

**Authors: Thank you. We evaluated the validity of the findings and results are shown in Appendix.**

**The AIC value of 7629.43 suggests that the model achieves a reasonable balance between fit and complexity. Furthermore, when compared to the null model, the reduction in the AIC value indicates that this model provides a better fit to the data.**

**The residual deviance of 7577.43 also points to a good fit of the model to the data. Its significance can be further assessed by comparing it to the null deviance, which helps determine the extent to which the model improves over a baseline model with no predictors.**

Null model:

• The adequacy of the sample size for a stable multinomial logistic regression model is a critical consideration, especially given the number of predictor variable categories. Adhering to the events per predictor parameter (EPP) rule ensures the statistical soundness and reliability of the findings. Harrell (2001, DOI: 10.1007/978-3-319-19425-7) suggests at least 15 EPP, and others recommend at least 20 EPP (e.g., Ogundimu et al., 2016, DOI: 10.1016/j.jclinepi.2016.02.031; Austin & Steyerberg, 2014, DOI: 10.1177/0962280214558972). Based on Table 1, adhering to the 15 EPP rule requires at least 345 events, but the smallest outcome category (i.e., diagnosed diabetes) has only 333 events. This should be discussed in the limitations section.

**Authors: Thank you for your wonderful suggestion. We added the following statement in the limitation:**

**The Events Per Predictor (EPP) rule is a widely accepted guideline used to determine the minimum number of events (observations) needed for each category of outcome variable to ensure the reliability of a multinomial logistic regression model. In this case, the sample size for the smallest outcome category (diagnosed diabetes, with 333 events) falls short of the recommended EPP threshold of 15, which would require at least 345 events. This discrepancy highlights a potential limitation in the model's stability and reliability.**

• The peer reviewer’s comment on this was not addressed in the revision. The statement, ‘We classified the wealth status as a household-level element as Poor, Middle, and Rich groups,” lacks clarity. Was this classification based on a specific reference or methodology? If so, please provide the source. If it was developed independently, additional justification and details are needed.

**Authors: Thank you. It is briefly explained in the manuscript with a reference.**

**In the BDHS, wealth status is assessed using a wealth index that classifies households into quintiles based on assets and amenities, such as ownership of consumer goods and housing characteristics [20]. This index is calculated through principal component analysis (PCA) to rank households into five categories: Poorest, Poorer, Middle, Richer, and Richest. For analysis, the poorest and poorer categories are combined as "poor," while the richer and richest are grouped as "rich," resulting in three overall wealth status categories.**

**USAID The DHS Program: Wealth Index Available from: https://dhsprogram.com/topics/wealth-index/**

• It is unclear whether a specific framework or rationale was followed for selecting predictors in the model. Did the authors rely on theoretical considerations, empirical evidence, or data-driven approaches (e.g., stepwise selection)? If so, please state this clearly and justify the choices made. Providing transparency here would enhance the study’s methodological rigor.

**Authors: We previously utilized a directed acyclic graph (DAG) – a visual representation of hypothesized causal relationships – to identify confounders in these relationships and the plot is given in the Appendix. In this DAG (detailed in Supplement, constructed with DAGitty: http://www.dagitty.net/dags.html#), diabetes status was considered the outcome, while residency was the main exposure. For the association between exposures and diabetes, a minimal sufficient adjustment set including age, sex, BMI, hypertension and residency was determined.**

Discussion

• The manuscript’s discussion section restates key findings and compares them to studies in agreement but offers limited mechanistic explanations for the observed patterns. Providing insights into the underlying biological, behavioral, or systemic factors contributing to these patterns would enhance the discussion.

**Authors: The discussion section is rewritten.**

• The manuscript highlights regional differences but does not explore the underlying causes of these disparities in depth. Evidence suggests that people living in coastal areas may experience a higher prevalence of diabetes than those in inland regions due to a combination of lifestyle, dietary habits, and environmental factors (DOI: 10.1136/bmjdrc-2015-000110; 10.1186/1471-2458-13-299; 10.15226/2374-6890/4/3/00181), which is not in line with the study findings. Expanding on these aspects would better align the discussion with the title’s focus.

**Authors: Thank you for your insightful comment. We have addressed this by including mosaic plots in the Appendix, and the corresponding findings are discussed in detail within the manuscript.**

**The plots depict the distribution of various demographic, health, and socioeconomic factors across residential regions (City, Rural, and Semi-Urban). Key observations include:**

- **Older populations (60+ years) are more concentrated in rural and semi-urban areas, likely due to aging in place and the migration of younger generations to urban centers.**
- **Urban areas exhibit a higher prevalence of hypertension, potentially driven by lifestyle factors such as consumption of processed foods, physical inactivity, and stress.**
- **The prevalence of obesity and overweight individuals is greater in urban regions, likely influenced by dietary habits, sedentary lifestyles, and socioeconomic status.**
- **In contrast, underweight individuals are more common in rural areas, possibly linked to lower income levels and nutritional deficiencies.**

**Regarding education:**

- **Higher education levels (college or above) are significantly associated with urban areas, likely due to better access to universities and educational institutions.**
- **Primary or no education is more prevalent in rural regions, highlighting disparities in access to educational resources.**
- **In terms of employment and income, urban areas have higher employment rates, reflecting greater job availability in industries, services, and corporate sectors.**
- **Rural areas show a higher proportion of individuals who are "not working," which may include agricultural laborers or unemployed individuals.**
- **Urban residents are more likely to fall into the "rich" category, reflecting better income opportunities and higher living costs.**
- **Rural areas have a higher percentage of "poor" individuals, consistent with limited job opportunities and lower wages.**

**These patterns underscore significant disparities in health, education, employment, and income across residential regions.**

• Overall, the whole discussion section requires a thorough review. Some examples:

a) The statement, "Interestingly, individuals with college education exhibit a lower risk for diagnosed diabetes but a higher risk for undiagnosed diabetes," should be revised because the RRRs for diagnosed diabetes are not statistically significant.

**Authors: Thank you. We have removed the sentence.**

b) Similarly, for undiagnosed diabetes, terms like “greater” should not be used when the 95% confidence intervals for overweight and obese categories overlap.

**Authors: Many thanks. We corrected it.**

c) The claim, “The analysis reveals a difference in diabetes prevalence based on residence type,” should be clarified or revised. While regional differences are evident, no significant differences by residence type (urban, rural, semi-urban) were found.

**Authors: Many thanks. We corrected it.**

d) To explore region/community-level factors, incorporating data from the community questionnaire, such as the distance to healthcare facilities, could provide valuable insights into regional disparities.

**Authors: Thank you for your insightful comment. Unfortunately, the dataset does not include information on the distance to healthcare facilities, which limits our ability to incorporate such community-level data into the analysis.**
